# Antioxidant and Antiproliferative Activities of Bioactive Compounds Contained in *Rosmarinus officinalis* Used in the Mediterranean Diet

**DOI:** 10.1155/2019/7623830

**Published:** 2019-11-16

**Authors:** Mohammed Bourhia, Fatima Ezzahra Laasri, Hind Aourik, Aicha Boukhris, Riaz Ullah, Ahmed Bari, Syed Saeed Ali, Mohammed El Mzibri, Laila Benbacer, Said Gmouh

**Affiliations:** ^1^Research Unit and Medical Biology, National Center for Nuclear Energy, Science and Technology (CNESTEN), Rabat 10001, Morocco; ^2^Laboratory of Chemistry, Biochemistry, Nutrition, and Environment, Faculty of Medicine and Pharmacy, University Hassan II, Casablanca, Morocco; ^3^Laboratory of Nutrition, Health and Environnement, Faculty of Science, Ibn Tofail University, Kenitra, Morocco; ^4^Faculty of Sciences Ben M'Sik, University Hassan II Casablanca, B. P. 7955, Casablanca, Morocco; ^5^Laboratory REMTEX, Higher School of Textile and Clothing Industries, Km 8, Route d'El Jadida, Casablanca, Morocco; ^6^Medicinal Aromatic and Poisonous Plants Research Center, College of Pharmacy, King Saud University, P. O. Box 2457, Riyadh 11451, Saudi Arabia; ^7^Central Laboratory, College of Pharmacy, King Saud University, P. O. Box 2457, Riyadh 11451, Saudi Arabia

## Abstract

**Background:**

*Rosmarinus officinalis (R. officinalis)* is a medicinal plant called rosemary, largely used in the Mediterranean diet for many decades ago.

**Objective:**

The aim of the present study was to investigate the polyphenolic content, the antioxidant activity, and the antiproliferative effect against human prostate cancer cell lines (LNCaP) of carnosol and carnosic acid as bioactive compounds contained in *R. officinalis* growing in Morocco.

**Materials and Methods:**

Polyphenolic content of *R. officinalis* ethanolic extract was studied using colorimetric assay. Carnosol and carnosic acid contained in *R. officinalis* extract were quantified using high-performance liquid chromatography (HPLC). The antiproliferative effect of the studied extracts on LNCaP was evaluated by WST-1 bioassay, and the antioxidant activity was assessed using DPPH assay.

**Results:**

The extracts of *R. officinalis* showed an important polyphenolic content ranging from 74.15 *μ*g·GAE/mg to 146.63 *μ*g·GAE/mg. The percentage of carnosol and carnosic acid in rosemary crops ranges from 11.7 to 17.3% and 1.09% to 3%, respectively. The extracts of *R. officinalis* exhibited a promoting antioxidant activity with IC_50_ ranging from 0.236 mg/mL to 0.176 mg/mL. Regarding the antiproliferative effect, the WST-1 assay revealed that all the tested extracts reduced notably the cell viability with IC_50_ values ranging from 14.15 to 15. 04 *μ*g/mL.

**Conclusion:**

In the current work, carnosol and carnosic acid exhibit antioxidant and antiproliferative activities in a concentration-dependent manner.

## 1. Introduction

Many people throughout the world use plants to treat diseases [1]. In Morocco, folk medicine has been spread all over the country. Ethnobotanical and ethnopharmacological surveys conducted in different Moroccan areas have shown more than 500 plant species used in Moroccan alternative medicine [2]. During the last decades, great efforts were drawn to characterize natural products derived from plants having biological activities for combating human diseases. Numerous phytochemical compounds have been identified and investigated for their therapeutic properties, including anti-inflammatory, anticarcinogenic, antiatherosclerotic, antibacterial, antifungal, anti-allergic, antiviral, and antimutagenic activities [3, 4].

*R. officinalis* (rosemary), a member of family Lamiaceae, is one of the most popular perennial culinary herbs. It is native to the Mediterranean region and cultivated worldwide. Especially, rosemary leaves are extensively used traditionally to treat numerous ailments. This plant has drawn more attention due to its several biological activities, including antihyperglycemic [5], antibacterial [6], anticancer [7], anti-inflammatory [8], antioxidant [9], antithrombotic [10], and hepato-protective effects [11]. In the food industry, rosemary extract is widely used as a safe and effective natural antioxidant [12]. Furthermore, rosemary has been largely incorporated in the Mediterranean diet [13].

In Morocco, *R. officinalis* is wildly growing in forests and in well-drained calcareous substrates. It is mainly spread in the Eastern Rif, Middle Eastern Atlas, Eastern High Atlas, and highlands of the Oriental Moroccan regions. This plant is adapted to grow in semiarid and subhumid bioclimatic regions at the level of thermo-Mediterranean and meso-Mediterranean vegetation [14].

Pharmacological activities of *R. officinalis* were essentially attributed to the presence of polyphenols like carnosic acid and rosmarinic acid [15]. These compounds are mainly responsible for antioxidative capacity and are implicated in molecular mechanisms associated with tumor inhibitory activities [16].

The current study was planned to assess the phenolic composition, to screen the bioactive molecules, to investigate the antioxidant power, and to evaluate the antiproliferative effect on the human prostate cancer cell lines of four Moroccon rosemary crops.

## 2. Materials and Methods

### 2.1. Plant Materials

**Figure 1:**
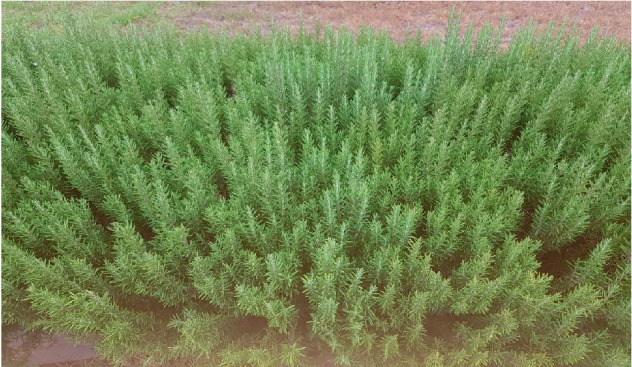
*Rosmarinus officinalis*.

**Figure 2:**
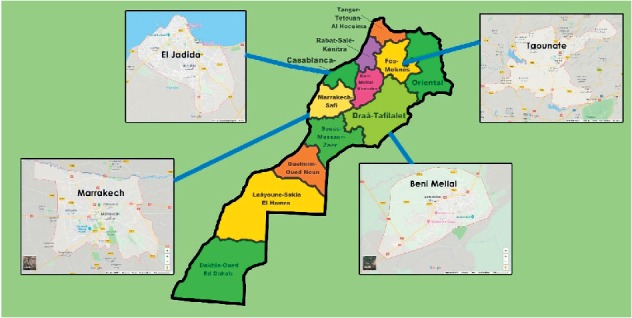
Map of Moroccan regions of plant's collection.

*R. officinalis* (Figure 1) was collected in June 2016 from different Moroccan regions: Marrakesh (31°38′2.98″N: −7°59′59.78″W), Taounate (34°32′11.80″N: −4°38′24.32″W), El Jadida (33°15′17.71″N: −8°30′21.67″W), and Beni Mellal (32°20′14.10″N: −6°20′59.39″W), as shown in Figure 2. The studied plant was identified by a taxonomist and has been deposited in the herbarium of Faculty of Sciences Ben M'Sik, University Hassan II. The leaves were dried and pulverized into a fine powder and then extracted with ethanol using maceration at room temperature. The obtained mixture was filtered and then evaporated under reduced pressure using a rotary evaporator to yield four extracts according to the plant collection area: rosemary extract collected from Marrakesh region (RM), rosemary extract collected from Taounate region (RT), rosemary extract collected from El Jadida region (RT), and rosemary extract collected from Beni Mellal region (RBM).

### 2.2. Phenolic Content

The total phenolic content was estimated by a colorimetric assay [17]. Briefly, 200 *μ*l of each extract was mixed with 1 ml of Folin–Ciocalteu reagent (1 : 10 V/V diluted with distilled water), allowed to stand at room temperature for 5 min, and then 800 *μ*l of sodium carbonate solution (75 g/L in water) was added. After 1 h of incubation, the absorbance was read at 760 nm. A standard calibration curve was plotted using gallic acid (0∼100 mg/L). Results were expressed as mg GAE (gallic acid equivalents) per gram of dry extract.

### 2.3. High-Performance Liquid Chromatography Analysis

The four dried extracts were dissolved in methanol, filtered, and then injected in HPLC (high-performance liquid chromatography) system. The column used in the current analysis was a Hypersil ODS C18 type (*l* = 125 mm; Φ = 4 mm, 5 *μ*m). The separation was isocratically undertaken with a mobile phase consisting of 0.5% of aqueous orthophosphoric acid, acetonitrile, and methanol (30 + 35 + 35) at a flow rate of 0.8 ml/min. In the HPLC analysis, the detection was done by UV-visible wavelengths due to the injection of 20 *μ*l. The retention time and chromatographic peaks of UV-Vis spectra of the checked compounds (carnosic acid and carnosol) were obtained and compared to reference standards at 205 nm.

### 2.4. Antioxidant Activity

In the present study, the antioxidant activity was evaluated using DPPH (2,2-diphenyl-1-picrylhydrazyl) assay [17]. Briefly, 200 *μ*L of various dilutions of extracts was mixed with 2.6 mL of methanolic DPPH solution (0.1%). After 30 min of incubation in darkness at laboratory temperature 25 ± 2°C, the radical scavenging activity was measured spectrophotometrically at 517 nm. In the current work, the ethanol solvent was used as a negative control. 3,5-di-tert-butyl-4-hydroxy-toluene (BHT) was used as a positive control. DPPH free radical scavenging percentage was calculated as follows:

(1) $$\begin{array}{c} \text{DPPH radical scavenging assay}\left(\%\right)=\frac{\left(\text{A blank}-\text{A test}\right)}{\text{A blank}}\times 100, \end{array}$$

where A blank is the absorbance of the control reaction. A test is the absorbance of the test compound.

For each plant extract, IC_50_ value (required concentration to reduce 50% of the initial population) was identified graphically from the nonlinear regression analysis.

### 2.5. Antiproliferative Effect

The antiproliferative effect of rosemary ethanolic extract was assessed on human prostate cancer cell lines (LNCaP). These cells were cultured in RPMI 1640 medium, supplemented with fetal calf serum (10%), antibiotics (1%), and glutamine (1%). The antiproliferative effect of the plants extracts collected from different Moroccan regions was evaluated using WST-1 (disodiummono {4 - [3 - (4 - iodophenyl)-2-(4-nitrophenyl)-2H-tetrazol]-3-ium-5-yl] benzene-1,3-disulfonate}) assay, based on the mitochondrial metabolic activity.

After the exponential growth of LNCaP cell lines seeded in 96-well microplates at a density of 8000 cells per well, the culture medium was removed and replaced with 100 *μ*l of fresh medium containing different concentrations of rosemary extracts ranging from 7.81 to 250 *μ*g/ml. After 48 h of incubation, the medium was removed again, and 10 *μ*l of WST-1 reagent was added in each well. Mitomycin was used as a positive control. The plates were incubated further for 4 h. Cell viability was assessed by reading the absorbance of each well at 450 nm using a Wallac Victor X3 multiplate reader.

The inhibitory percentage was defined according to the following equation:

(2) $$\begin{array}{c} \text{cell death}\left(\%\right)=\frac{\text{control OD}-\text{sample OD}}{\text{control OD}}*100. \end{array}$$

For each extract, IC_50_ value (concentration required to reduce 50% of the initial population) was determined graphically from the regression curve performed on WST-1 assay viability.

### 2.6. Statistical Analysis

Quantitative data were expressed as the average of triplicate experiments ± SD (standard deviation). The significance of differences between the means was evaluated using ANOVA. The means were pairwise compared using the Tukey–Kramer multiple comparisons test. Statistically, a significant difference was considered at *P* < 0.05.

## 3. Results

### 3.1. Polyphenolic Content

**Figure 3:**
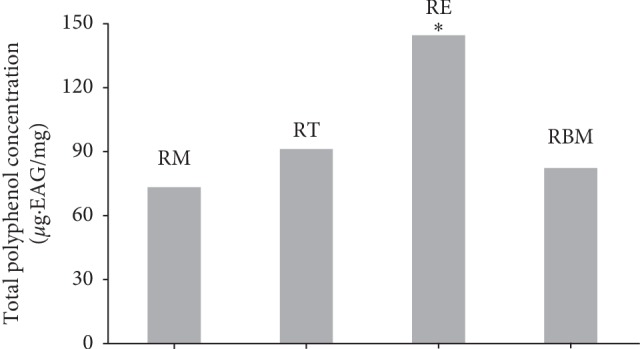
Phenolic content of ethanolic extracts prepared from *R. officinalis* growing in different Moroccan regions (Marrakesh (RM), Taounate (RT), El Jadida (RE), and Beni Mellal (RBM)).

The ethanolic extracts prepared from rosemary growing in four different Moroccan regions were evaluated for their polyphenolic content. The results showed that the total polyphenolic content was very important in plants harvested from different areas; however, the ethanolic extract prepared from the plant collected from the El Jadida region showed the highest amount of polyphenols compared to other rosemary crop areas (*P* < 0.05) (Figure 3).

The total polyphenolic content contained in plants collected from each area such as El Jadida, Taounate, Beni Mellal, and Marrakesh was estimated at 146.63 *μ*g·GAE/mg, 92.39 *μ*g·GAE/mg, 83.27 *μ*g·GAE/mg, and 74.15 *μ*g·GAE/mg, respectively. No significant difference in polyphenolic content was recorded between the rosemary crops growing in Marrakesh, Taounate, and Beni Mellal. (*P* > 0.05). The only significant difference was registered between El Jadida crop and the other crops (*P* < 0.05).

### 3.2. High-Performance Liquid Chromatography Analysis

**Figure 4:**
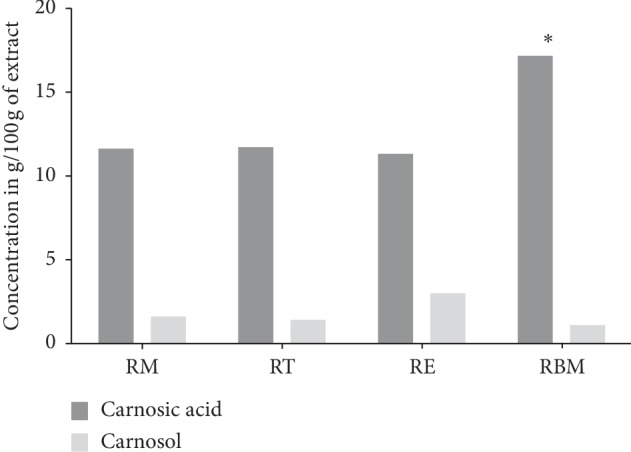
Carnosic acid and carnosol content in rosemary ethanolic extract (results are given in %). (Marrakech rosemary (RM), Taounate rosemary (RT), El Jadida rosemary (RE), and Beni mellal rosemary (RBM)).

HPLC analysis of the rosemary extracts led to separation, identification, and quantification of both carnosic acid and carnosol. The identification of these compounds was carried out by comparing retention times to standard compounds (Figure 4).

**Figure 5:**
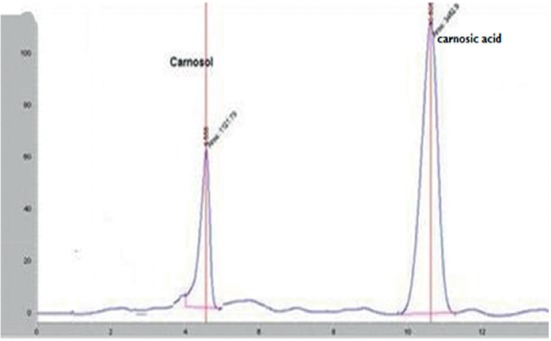
HPLC chromatogram of rosemary extract growing in Marrakesh land.

**Figure 6:**
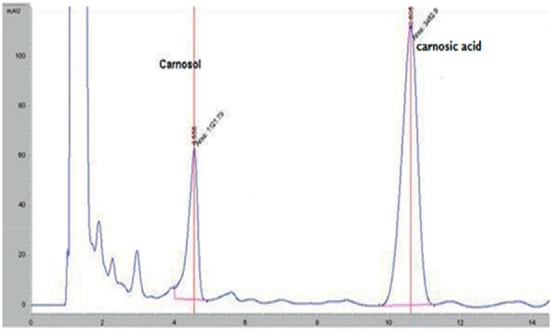
HPLC chromatogram of rosemary extract growing in Taounate land.

**Figure 7:**
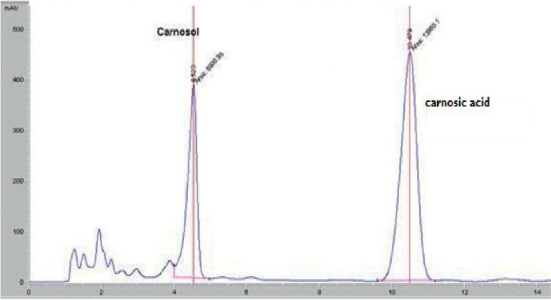
HPLC chromatogram of rosemary extract growing in El Jadida land.

**Figure 8:**
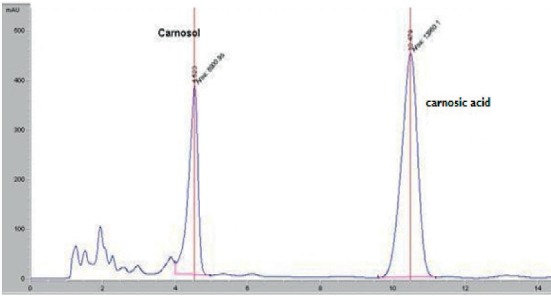
HPLC chromatogram of rosemary extract growing in Beni Mella land.

HPLC separation profile revealed the presence of two major chromatographic peaks of carnosol and carnosic acid in all studied rosemary crops. Both carnosol and carnosic acid presented at significant content in different rosemary crops (Figures 5–8). The percentage of carnosic acid and carnosol dosed in plants harvested from all areas ranges from 11.7 to 17.3% and 1.09% to 3%, respectively.

Regarding the carnosic acid, no significant difference was observed between the rosemary crops growing in Marrakesh, Taounate, nor El Jadida (*P* > 0.05); however, the only significant difference was reported between Beni Mellal crop and the other studied crops (*P* < 0.05). Concerning the carnosol compounds, the three studied crops such as Marrakesh, Taounate, and Beni Mellal showed no significant difference in carnosol content (*P* > 0.05); meanwhile, a high significant was remarked between El Jadida crops and the other crops investigated in the current work (*P* < 0.05).

The great concentration of carnosic acid was found in extracts of rosemary growing in Beni Mellal land, whereas the carnosol prevailed in the extract of rosemary growing in El Jadida land. The plants growing in Marrakesh and Taounate lands have approximately similar content of carnosic acid and carnosol.

### 3.3. Antioxidant Activity

**Figure 9:**
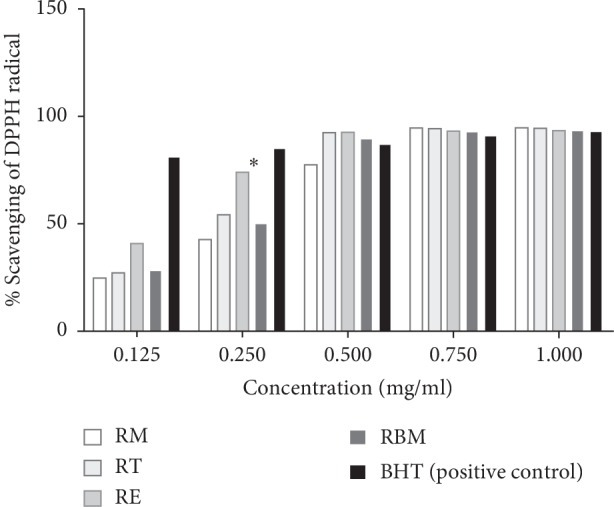
DPPH radical-scavenging activities of ethanolic extract of rosemary growing in different Moroccan lands (Marrakesh rosemary (RM), Taounate rosemary (RT), El Jadida rosemary (RE), and Beni Mellal rosemary (RBM)).

All rosemary plants growing in the four Moroccan lands showed interesting antioxidant activities in a dose-dependent manner. DPPH IC_50_ of rosemary ethanolic extracts prepared from plants collected from each area such as El Jadida, Taounate, Beni Mellal, and Marrakesh was estimated at 0.302 mg/mL, 0.258 mg/mL, 0.236 mg/mL, and 0.176 mg/ml, respectively (Figure 9). The extract of rosemary growing in El Jadida land exhibited the most antioxidant power compared to plants growing in the other regions. The ethanolic extract of rosemary harvested from the El Jadida area exhibited the lowest DPPH IC50 and the highest phenolic content. The extracts studied in the present work exhibited DPPH radical scavenging in a concentration-dependent manner.

Regarding the antioxidant activity, the analysis of variance showed a significant difference between El Jadida crop and the other studied crops especially with lower concentration (0.25 mg/ml) (*P* < 0.05).

### 3.4. Antiproliferative Effect

In the current work, the antiproliferative effect of ethanolic extracts of *R. officinalis* growing in the four different Moroccan lands was tested on human prostate cancer cell lines (LNCaP) using WST-1 colorimetric assay.

**Figure 10:**
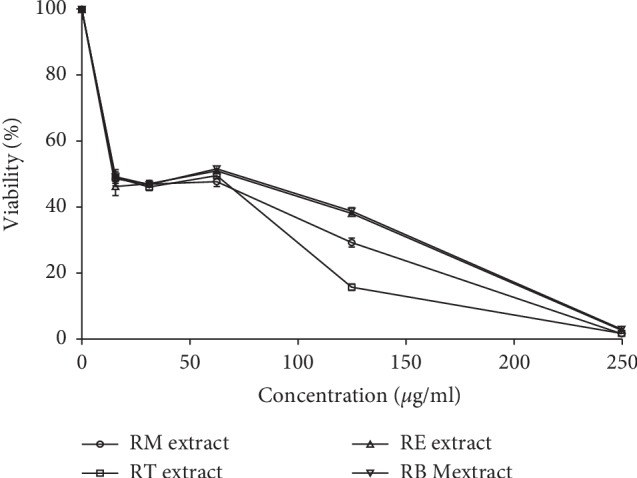
Antiproliferative effect of rosemary ethanolic extracts on the prostate cancer cell lines (LNCaP) after 72 h of treatment (Marrakesh rosemary (RM), Taounate rosemary (RT), El Jadida rosemary (RE), and Beni Mellal rosemary (RBM)).

The WST-1 test revealed that the ethanolic extracts of *R. officinalis* collected from different areas in Morocco which tested in a concentration ranging from 0 to 250 *μ*g/mL reduced significantly the cell viability of human prostate cancer cells lines (LNCaP) (Figure 10).

IC_50_ value (the inhibitory concentration required to inhibit 50% of the initial population) of rosemary collected from the studied areas such as El Jadida, Taounate, Beni Mellal, and Marrakesh was determined approximately at 14.15 *μ*g/mL, 14.93 *μ*g/mL, 14.95 *μ*g/mL, and 15.04 *μ*g/mL, respectively. All crops of rosemary reduced the cell viability of the human prostate cancer cell lines (LNCaP) in a concentration-dependent manner. The analysis of variance showed no significant difference between IC_50_ of rosemary crops on the tested cancer cell lines (*P* > 0.05). Hence, an equal antiproliferative effect on the human prostate cancer cell lines (LNCaP) was registered for all investigated rosemary crops in the current research study.

## 4. Discussion

The present work focused on the study of *R. officinalis* growing in different Moroccan lands: Taounate land located in the mountainous Rif region, Beni Mellal in the mountainous Atlas region, Marrakesh in the southeast of Morocco, and El Jadida at the coastal plain (Figure 2). These regions are completely different in terms of climatic conditions and edaphic characteristics. They are able to affect the chemical composition of plants and, therefore, the biological activities [18].

The current work was conducted to screen the polyphenol content, carnosol, carnosic acid, antioxidant activity and antiproliferative effect of Morrocan rosemary crops. The phenolic content in extracts of *R. officinalis* was well documented as reported in the current work. On the other hand, numerous biological activities are related to the polyphenolic composition of plants like antioxidant power [19]. The tested extracts were highly rich in carnosol and carnosic acid. These polyphenolic compounds are known for their antioxidant and antitumoral properties [16]. The phenolic diterpenes like carnosol and carnosic acid are strongly responsible for radical oxygen species scavenging as reported in previous studies [20]. In the present work, the antioxidant activity shown in rosemary ethanolic extract is frequently due to its carnosol and carnosic acid content [21–23]; thus, the carnosol and carnosic acid revealed in the studied extract exhibit an antioxidant activity in a concentration-dependent manner.

It was very important to screen the carnosol and carnosic acid in rosemary extracts in order to evaluate their probable relationship with the detected activities, such as the antioxidant activity and the antiproliferative effect on a human prostate cancer cell lines (LNCaP) investigated in the current work. Our results were in accordance with those reported in earlier literature [24, 25]; it was reported that carnosic acid and carnosol are the major bioactive constituents contained in rosemary leaf polar extract [26]. *R. officinalis* contains other chemicals such as rosmarinic acid, luteolin, apigenin, carnosol, caffeic acid, and scutellarin presented at a lower concentration [24, 27]. In the current work, rosemary collected from each geographical location, such as Marrakesh, Taounate, El Jadida, and Beni Mellal, contained a remarkable amount of carnosol and carnosic acid with drawing attention to plant collected from El Jadida land which showed to contain higher content of these compounds compared to plants collected from the other regions. It was reported that a synergistic effect takes place between carnosol, carnosic acid, and rosmarinic acid for enhancing the antioxidant power of the studied extracts [28]. Rosemary derivatives can establish a synergic effect with several antitumor agents in order to solidify their anticancer action [16].

*R. officinalis* is a sensitive plant to pedoclimatic variations that could affect the chemical composition of plants. In the present study, the rate of carnosol and carnosic acids was found to be different between the studied rosemary crops. These results were in concordance with previous data [18, 29, 30], in which it was highlighted that the chemical composition of plants is strongly associated and influenced by the environmental conditions. As reported in the current work, the rosemary plants collected from each geographical location such as Marrakesh, Taounate, El Jadida, and Beni Mellal contained a remarkable amount of carnosol and carnosic acid with drawing attention to plant collected from El Jadida land which showed to contain higher content of these compounds compared to plants collected from the other regions. The climate conditions were responsible for the variation of plant composition as reported in the present work. These findings were used for performing a comparison with previous data [30], in which it was reported that the methanolic extracts of *R. officinalis* collected from three different regions showed different antioxidant activity according to the plant collection area.

Rosemary and their derivative compounds have been widely investigated for their antiproliferative properties against cancer cell lines [31]. As reported in previous studies, *R. officinalis* extracts reduced remarkably the cell viability of gastric and esophageal carcinoma cell lines [32], colon adenocarcinoma cell lines [33, 34], and cervical epithelial carcinoma cell lines [35]. The carnosic acid dosed in the studied plant in the current work exerted a significant antiproliferative effect on prostate cancer cell lines [36]. Thus, carnosol and carnosic acid exerted the antiproliferative effect in a concentration-dependent manner. The probable mechanism of actions of rosemary extracts involved in cancer cell death could be due to inducing mitochondrial-dependent apoptosis, in which the plant's extracts increased the expression of the proapoptotic protein Bax and decreased the expression of the antiapoptotic Bcl-2 protein as described in earlier literature [36]. Modulation of the caspase pathway and inhibition of phosphatidylinositide-3 kinase (PI3K)/Akt signaling pathway were also reported as a mechanism of action of rosemary derivatives involved in cancer cell death [36]. The antiproliferative effect of carnosol on prostate cancer cell lines could be also due to inducing dysregulation of multiple signaling pathways, such as downregulation of Bcl-2 and procaspase-8 and upregulation of Bax2 [37]. *R. officinalis* crude extract and its derivative compounds like carnosic acid, carnosol, and rosmarinic acid have been largely used as phytochemical supplements for cancer prevention and treatment [38].

## 5. Conclusion

The present report gives big data on rosemary plants growing in different geographical zones of Morocco, namely, Marrakesh, Taounate, El Jadida, and Beni Mellal in terms of phytochemical content and antioxidant and antiproliferative effects. The studied plant in the current work represents a promising source of high antioxidant and antiproliferative chemicals. In highlight of the obtained results, we could confirm that the best rosemary crops in terms of carnosol and carnosic acid, as well as the studied activities, were El Jadida and Beni Mellal rosemary crops. Therefore, the Moroccan rosemary plants especially those growing in Jadida and Beni Mellal regions could be interesting for conceptualizing new drugs that are effective against cancer and free radical-mediated diseases.

## Data Availability

The data used to support the findings of this study are included within the article.
